# Functional MRI in the Investigation of Blast-Related Traumatic Brain Injury

**DOI:** 10.3389/fneur.2013.00016

**Published:** 2013-03-04

**Authors:** John Graner, Terrence R. Oakes, Louis M. French, Gerard Riedy

**Affiliations:** ^1^National Intrepid Center of Excellence, Walter Reed National Military Medical CenterBethesda, MD, USA; ^2^National Capital Neuroimaging Consortium, Uniformed Services University of the Health SciencesBethesda, MD, USA; ^3^Walter Reed National Military Medical CenterBethesda, MD, USA; ^4^Center for Neuroscience and Regenerative MedicineBethesda, MD, USA; ^5^Defense and Veterans Brain Injury Center, Walter Reed National Military Medical CenterBethesda, MD, USA

**Keywords:** blast injuries, traumatic brain injury, review, military injury, functional magnetic resonance imaging

## Abstract

This review focuses on the application of functional magnetic resonance imaging (fMRI) to the investigation of blast-related traumatic brain injury (bTBI). Relatively little is known about the exact mechanisms of neurophysiological injury and pathological and functional sequelae of bTBI. Furthermore, in mild bTBI, standard anatomical imaging techniques (MRI and computed tomography) generally fail to show focal lesions and most of the symptoms present as subjective clinical functional deficits. Therefore, an objective test of brain functionality has great potential to aid in patient diagnosis and provide a sensitive measurement to monitor disease progression and treatment. The goal of this review is to highlight the relevant body of blast-related TBI literature and present suggestions and considerations in the development of fMRI studies for the investigation of bTBI. The review begins with a summary of recent bTBI publications followed by discussions of various elements of blast-related injury. Brief reviews of some fMRI techniques that focus on mental processes commonly disrupted by bTBI, including working memory, selective attention, and emotional processing, are presented in addition to a short review of resting state fMRI. Potential strengths and weaknesses of these approaches as regards bTBI are discussed. Finally, this review presents considerations that must be made when designing fMRI studies for bTBI populations, given the heterogeneous nature of bTBI and its high rate of comorbidity with other physical and psychological injuries.

## Summary

This review focuses on the application of functional magnetic resonance imaging (fMRI) to investigate blast-related traumatic brain injury (bTBI). Relatively little is known about the exact mechanisms of neurophysiological injury and pathological and functional sequelae of bTBI. Furthermore, in mild bTBI, standard anatomical imaging techniques [MRI and computed tomography (CT)] generally fail to show focal lesions and most of the symptoms present as subjective clinical functional deficits. Therefore, an objective test of brain functionality has great potential to aid in patient diagnosis and provide a sensitive measurement to monitor disease progression and treatment. The heterogeneity of injury in the bTBI population and resulting lack of a specific anatomical target present a challenge when attempting to develop an fMRI protocol sensitive to TBI in blast victims. The goal of this review is to present the relevant body of blast-related TBI literature and, combined with a brief review of some currently used fMRI techniques, present suggestions and considerations in the development of bTBI fMRI studies.

## bTBI Occurrence

Traumatic brain injury following blast exposure is quite prevalent in soldiers returning from the conflicts in Iraq and Afghanistan. Although there is a range of estimates regarding the occurrence of TBI and bTBI in the recent military operations (Tanielian and Jaycox, 2008), the Joint Theater Trauma Registry has reported that, of the soldiers seen at Landstuhl Regional Medical Center, 22% had head, face, or neck injuries (Okie, 2005). The Armed Forces Health Surveillance Center reports that between January 2003 and January 2010, approximately 135,000 military service members were diagnosed with TBI, most of them mild^1^. One study (Murray et al., 2005) found that 78% of injuries seen in one combat field setting were due to explosions. The mechanism of injury for 87.9% of the service members with traumatic limb amputation was some form of explosive device (Stansbury et al., 2008). Another study (Hoge et al., 2008) reported that in those sustaining TBI with loss of consciousness (LOC), blast was involved in 79% of the injuries. While explosions were also a significant source of injury in World War I and World War II, accounting for 35 and 73% of the injuries, respectively, injuries sustained from explosions accounted for 78% of Operation Iraqi Freedom (OIF)/OEF injuries, the highest proportion seen in any large-scale conflict (in contrast to gunshot wounds which accounted for just 18% of the injuries from 2001 to 2005; Owens et al., 2008).

The relative numbers of TBI and bTBI cases seen in wounded military personnel returning from modern conflicts are larger than those seen in previous wars (Warden et al., 2009). This is most likely due to several factors, including advances in protective equipment (for example, thoracic armor reducing the potential for fatal pulmonary damage due to blast exposure) and in-the-field medical capabilities (Okie, 2005; Bass et al., 2012; Wood et al., 2012).

While the incidence of occurrence has risen sharply within the previous few years, there is a relatively limited body of research in blast-related TBI. A PubMed^2^ literature search using the keywords “traumatic brain injury” yielded 62,323 results, of which 25,616 (41%) were published after March 20, 2003, the beginning of OIF. By contrast, literature searches for articles containing the keywords “blast traumatic brain injury” or “explosive traumatic brain injury” or “explosion traumatic brain injury” yielded only 524 results, 387 of which were published after March 20, 2003. There may be several factors contributing to the relative scarcity of blast-related TBI literature. The relative levels of disparity and similarity between blast-related TBI and other forms of TBI (blunt trauma, etc.) are not well resolved. A lack of solid classification of blast-related TBI as a unique form of injury may have led to fewer blast-specific research studies. Finally, the relatively lower occurrence or observation of blast-related TBI seen prior to current military conflicts may also have contributed to a low focus on bTBI research.

## Summary of Recent bTBI Publications

Several recent bTBI-related publications describe research-related and clinical challenges currently presented by bTBI. Recent blast-related animal studies have attempted to produce accurate exposure-injury models in several species (for example, Rafaels et al., 2011, 2012). One major challenge facing such models is the ability to scale exposure-injury relationships between species in order to provide accurate predictive information regarding human blast exposure (Bass et al., 2012). Ling et al. (2009) review a number of current clinical approaches for treating bTBI, highlighting many of the physical sequelae that accompany bTBI as well as methods to address them. These authors comment that a greater understanding of the physical and neurophysiological bases of bTBI is needed along with a reliable diagnostic tool for clinical evaluation.

### Combat-related TBI

For those with combat-related TBI, clinical issues can be significant. French and Parkinson (2008) describe the medical complexity in these cases, with three clinical military bTBI composite case studies providing examples of the variety and complexity of bTBI sequelae. In one case, a patient’s increased irritability led to significant behavioral changes, including involvement in an increased number of verbal arguments. All three of the individuals included in the paper reported problems with short-term memory and attention or concentration. Overall, the case reports show how polytrauma and high rates of medical comorbidity can affect the diagnostic and treatment process. In the VA polytrauma system of care, Service Members injured by blast had a wider range of physical injuries, higher levels of opioid analgesic use upon admission and discharge, reduced improvement in pain intensity after treatment, and much higher rates of psychiatric diagnoses than those injured in combat via motor vehicle collisions, falls, or other mechanisms (Clark et al., 2009).

In combat-related TBI, the emotional context in which injury occurs also matters. Service Members are routinely exposed to emotionally traumatic events at high rates (Hoge et al., 2004). For those individuals that do sustain a mild TBI with LOC, there are high rates of comorbid post-traumatic stress disorder (PTSD; Hoge et al., 2008; Rosenfeld and Ford, 2010). It has been suggested (Kennedy et al., 2007) that damage to the pre-frontal cortex in TBI may result in disruption of neural networks involved in the regulation of anxiety, making the affected individual more vulnerable to the effects of an emotionally traumatic event. Whether or not this hypothesis may be relevant to blast-related TBI is unknown, however. Further, TBI has been shown to complicate or prolong recovery from preexisting or comorbid conditions such as PTSD (Vanderploeg et al., 2009). The complex problems facing care-givers of these TBI/PTSD patients are reviewed by Capehart and Bass (2012), who recommend an interdisciplinary approach to treatment. Previous studies (Lippa et al., 2010; Wilk et al., 2010; Belanger et al., 2011; Luethcke et al., 2011) have generally found no differences between blast and non-blast TBI on a range of symptoms such as depression, alcohol, happiness, vigor, fatigue, restlessness, anxiety, anger, and postconcussion symptoms. However, some studies have shown a relation between blast-related injury and PTSD symptom reporting (Sayer et al., 2008; Belanger et al., 2009, 2011; Kennedy et al., 2010; Lippa et al., 2010).

### Application of modern techniques to bTBI diagnosis

Several authors have reviewed the use of neuroimaging techniques for diagnosing bTBI. Benzinger et al. (2009) presented a discussion summary of a 2008 bTBI neuroimaging workshop in St. Louis. This report discusses how PET, CT, and MRI techniques may be applied to best address bTBI from a clinical standpoint as well as how these techniques may be incorporated into animal experiments. Van Boven et al. (2009) present a review article summarizing current and previous uses of various imaging techniques in the investigation of TBI and PTSD. In this work, the authors postulate that a wide range of MRI and PET imaging methods including diffusion tensor imaging, fMRI, MR spectroscopy, arterial spin labeling, susceptibility weighted imaging, FDG/metabolism imaging, and amyloid imaging may be of future use regarding both blast-related and non-blast-related TBI.

Initial attempts to diagnose and differentiate bTBI from non-blast TBI using individual imaging and clinical modalities have lead to disappointing results. When comparing blast to non-blast TBI, Belanger et al. (2010) saw no statistical difference in neurocognitive tests. A PET study on 12 mild bTBI demonstrated subtle hypometabolism in the cerebellum when compared to normals (Peskind et al., 2011). Mac Donald et al. (2011) and Hayes et al. (2012) show changes in DTI imaging but these reports suffer from the common theme of definition of a blast injury.

### Common themes

Despite the range of topics and views, several common threads run through current bTBI literature. First, the development of a more robust understanding of the mechanical effects primary blast-waves have on the human brain and how these effects then contribute to neurophysiological sequelae is necessary for an understanding of blast-related TBI. Secondly, there is a wide range of heterogeneity across bTBI victims, with respect to both characteristics of the causative incident as well as the course of disease progression and symptom manifestation. Thirdly, there is a need for reliable clinical tools to aid in the diagnosis and treatment planning of blast victims. The increasing availability and relative non-invasive nature of medical imaging techniques make them prime candidates for fulfilling this need. In addition, fMRI, which indirectly measures brain activation during functional tasks, may be more sensitive to damage to neural networks caused by TBI. One common theme seen in the clinics at Walter Reed is the increased cognitive effort reported by TBI patients. They often describe increased concentration and effort required to perform tasks that they could do “without thinking” prior to injury. While some of these symptoms could be related to comorbid states (as described by Capehart and Bass, 2012), sensitive diagnostic tests to evaluate and quantify the reported symptoms are needed. Unfortunately, the battery of neurocognitive testing performed is often normal in patients who describe this increased cognitive effort, suggesting that the tests might not be sensitive enough to detect the increased neural load requirements. Imaging of functional neural networks with fMRI has the potential to fill this gap. This review focuses on the challenges and considerations when applying one of these neuroimaging techniques, functional MRI, to a bTBI population.

## Blast-Related Brain Injury Mechanisms

The multiple physical aspects of exposure to a blast can create a variety of specific injury mechanisms. Primary damage may be caused as the blast-wave propagates through the brain and interacts with the surrounding skull. The exact mechanisms of this type of damage are not fully understood. There is currently a widening body of literature focused on the development of computational and physical models for determining the damaging forces experienced by the brain as a blast-wave moves through it (Moore et al., 2009; Chafi et al., 2010; Nyein et al., 2010; Alley et al., 2011; Nakagawa et al., 2011; Ganpule et al., 2012; Jerusalem and Dao, 2012; Panzer et al., 2012). Results from such models may indicate regions of the brain that are more susceptible to blast-wave damage, providing guidance for which brain regions and related brain functions to investigate with neuroimaging techniques or as additional support for the imaging findings.

An unfortunate element of bTBI is the almost universal co-occurrence of other injury mechanisms beyond the primary blast-wave. Current literature decomposes the effects of the blast TBI event on the brain into as many as four categories (DePalma et al., 2005; Benzinger et al., 2009; Cernak and Noble-Haeusslein, 2010; Ling et al., 2009). Primary effects are those due to the blast-wave itself as it travels through the brain tissue. Secondary blast effects are those inflicted by objects hurled or otherwise set in motion by the blast-wave which then strike the head, resulting in blunt or penetrating trauma. The tertiary effects of a blast are those caused by the violent movement of the victim due to the blast. These can be especially devastating in the case of an enclosed environment, such as the cabin of a vehicle (Leibovici et al., 1996; Taber et al., 2006; Ling et al., 2009). Finally, some authors have identified quaternary bTBI effects due to other damaging elements associated with the nature of the explosive device used, such as radiation, heat, or the release of chemicals (DeWitt and Prough, 2009). In selecting “blast-injured” TBI subjects for investigations, it is typical to include those with secondary and tertiary effects of blast (sometimes referred to as “blast-plus,” i.e., Moore and Jaffee, 2010; Lange et al., 2012). This is largely due to the rarity of documented isolated primary blast in this population. Given this, it is to be expected that individuals will have some injury characteristics related to the exposure to the primary blast, and some related to more traditional, mechanical injury.

### Secondary injury

Blunt impacts to the head from either secondary or tertiary effects can directly lead to contusions in the brain. Such impacts can produce violent, sudden movement of the brain within the skull, also causing diffuse axonal injury (DAI) or hemorrhage. In addition to these direct effects, the injury may lead to secondary injury, such as the creation of regions of edema or ischemia in the brain (Margulies and Hicks, 2009; Svetlov et al., 2009).

### Effects of systemic injury

Along with direct damage to the brain from the elements of the blast, damage to other organs of the body may augment the potential for brain injury. For example, exposure of the torso, if unprotected, to a primary blast-wave or objects hurled by a blast can injure the lungs. This can lead to a state of hypoxemia that presents an additional challenge to the neural system beyond the direct blast effects (Ritenour and Baskin, 2008; DeWitt and Prough, 2009). Blast victims may also sustain injuries such as limb loss leading to hemorrhage, hypovolemia, decreased brain perfusion, and hypotension. Such situations present challenges to the brain as a whole, not only to those areas directly affected by the blast.

Even if the head itself is shielded from the blast directly, there is evidence that blast impact to the lungs can lead to damage and biochemical alterations in the brain (Cernak et al., 1996, 2001). One potential mechanism for this is the kinetic transfer of energy from the lungs to the brain and central nervous system. The blast-wave overpressure may be passed through the vasculature in the lungs to that in the brain, allowing pressure-related injury to occur. Another potential method through which damage to the lungs may impact the brain is the creation of air emboli at the alveoli (Ho, 2002). These emboli may then pass through the vasculature and lead to ischemic conditions in the brain (Mayorga, 1997; Wolf et al., 2009). Recent work by Wood et al. (2012) suggests that modern body armor may greatly decrease the risk of pulmonary injury due to primary blast-wave exposure, thus limiting the potential for related systemic insult. However, this method of injury remains a possibility for unarmored blast victims.

### White matter damage

One of the consequences often seen in blast-related and non-blast-related TBI is the degeneration of white matter neurons (Bauman et al., 2009; Cernak and Noble-Haeusslein, 2010). This damage is most commonly seen in parasagittal white matter, the corpus callosum, and the brain stem (Meythaler et al., 2001; Povlishock and Katz, 2005). Hypotheses about the mechanism by which diffuse white matter damage occurs have changed as more research has been undertaken. The cell loss most likely involves both necrotic and apoptotic processes and may continue over a period of time following the injury (Povlishock and Katz, 2005). Such white matter injury has the potential to disrupt communication between parts of cognitive networks. This disruption may in part lead to some of the cognitive deficits seen in TBI victims (MacDonald et al., 2008; Strangman et al., 2009; Levin et al., 2010), even in cases where there are no regions of detectable gray matter damage (Scheid et al., 2006).

## Elements of bTBI

### Injury classification

Subjects with closed-head TBI are usually broken down into three classifications of injury severity: mild, moderate, and severe. A fourth category of TBI, penetrating TBI, is used when an object penetrates the dura and enters the brain. In military TBI, this is most commonly a bullet or metallic fragment. Classification of closed-head TBI is generally made via a combination of Glasgow Coma Scale score (GCS; Teasdale and Jennett, 1974), estimated duration of post-traumatic amnesia (PTA), and estimated duration of LOC at the time of injury (Kay et al., 1993; Maas et al., 2008). An individual with mild TBI is defined by the American Congress of Rehabilitation Medicine (ACRM; Esselman and Uomoto, 1995) as a

“… person who has had a traumatically induced physiological disruption of brain function, as manifested by at least one of the following: any period of loss of consciousness, and loss of memory for events immediately before or after the accident, any alteration of mental state at the time of the accident, and/or focal neurological deficits that may or may not be transient, so long as the severity of the injury does not exceed 1) loss of consciousness for more than 30 minutes, 2) after 30 minutes, a Glasgow Coma Scale score of 13-15, and 3) a period of post-traumatic amnesia not to exceed 24 hours.”

Additional categorization has also been proposed via structural findings in clinical MRI and CT readings (Marshall et al., 1992; Firsching et al., 2001). The presence or absence of focal lesions on MRI or CT is often used in patient participant discrimination for research studies and has been suggested in multiple publications as a potential reason for conflicting functional study results (Levine et al., 2006; Sanchez-Carrion et al., 2008b). While the degree to which these various characteristics ultimately affect group heterogeneity has not been fully elucidated, there is a general consensus of their importance when considering patient populations. As TBI severity is based on injury characteristics, rather than extent of longer-term symptom expression (i.e., headache, balance problems, memory problems, poor concentration, etc.), some individuals may be classified as having mild TBI and have significant persisting symptoms while others with a more severe TBI classification may have fewer symptoms. In one recent military TBI sample (Belanger et al., 2010) those with mild TBI reported more post concussive symptoms than a group of those with moderate to severe TBI, although this appeared to reflect level of post-traumatic stress in the population. In general however, increased severity of TBI is more likely to result in more persisting symptoms.

### Time since injury

An important factor for a TBI patient is the time since injury. While very few longitudinal TBI imaging studies have been reported, several groups have reported functional- or diffusion-related changes within TBI populations between initial and follow-up scan sessions, both with and without cognitive training in the interim (McAllister et al., 2006; Sanchez-Carrion et al., 2008a; Kim et al., 2009). Diffusion differences have also been noted between mild TBI subjects imaged less than 3 months post-injury and those imaged more than 3 months post-injury (Rutgers et al., 2008). These neuroimaging results and general rehabilitation outcomes for patients with TBI suggest a significant degree of change over time within individual subjects. Therefore, specific attention must be paid to differences in time from injury, even on the order of a few months, when creating TBI patient study populations. These differences in time since injury may correspond to significant differences in states of injury or recovery.

### History of TBI

History of prior TBI can also have an effect on a patient’s ability to recover following a subsequent TBI event. While the exact mechanism of this compounding effect is not yet known, evidence suggests that patients with previous TBI are at greater risk for suffering another TBI incident, may have greater cognitive symptom severity following another TBI incident, and may have prolonged recovery periods following another TBI incident (Gronwall and Wrightson, 1975; Gaetz et al., 2000; Guskiewicz et al., 2003). This seems to be particularly the case when the second TBI is in close temporal proximity to an earlier incident (Vagnozzi et al., 2007, 2008; Fujita et al., 2012). While this evidence is from animal studies and patients who experienced non-blast-related TBI, this compounding injury effect should be taken into consideration when creating bTBI neuroimaging study populations as well, as previous TBI may be common (Ommaya et al., 1996; Ivins et al., 2003).

### Behavioral symptoms

Clinical symptoms of bTBI patients vary dramatically from coma to mild persistent headache. Some of the commonly reported symptoms associated with mild bTBI include problems with working memory and selective attention, and feelings of mental fatigue (Dikmen et al., 1986; Alexander, 1995; Bate et al., 2001; Vanderploeg et al., 2001). The majority of these symptoms are functional in nature, making them potential targets for fMRI investigation.

## Relevance of Non-Blast TBI Findings to Blast-Related TBI

As the literature findings in the previous section show, much more research has been focused on the investigation of non-blast TBI than blast-related TBI. It remains unclear, however, how relevant non-blast TBI findings are to blast-related TBI (Elder et al., 2010). Both may share some aspects of injury causation, including blunt trauma and shearing strain on brain tissue. This may then in turn lead to shared forms of injury, such as hemorrhage and DAI. However, because the precise mechanics of neural injury due to a primary blast-wave are not fully understood, the degree to which the blast may affect or bolster injury caused by other methods of trauma is not known. In addition, systemic challenges due to the blast’s effects on other major organs of the body may offer further complications not typically seen in non-blast TBI. Blast victims may also suffer from increased probability of sensory impairment, post-injury pain, and polytrauma (French, 2010). These differences in acute injury may lead to differences in general underlying injury characteristics and recovery between non-blast TBI patients and bTBI patients. The potential for such differences should be taken into consideration when attempting to evaluate the degree to which blast- and non-blast TBI, and their corresponding bodies of literature, relate. Although differences in injury characteristics likely exist, there are also likely important areas of overlap between blast and non-blast injury mechanisms, injury sequelae, and associated research findings. Certainly from a neurocognitive standpoint, no differences have been found in neuropsychological evaluations between groups of service members sustaining TBI through a blast-related mechanism versus another mechanism (Belanger et al., 2009; Lange et al., 2012).

Similarly, brain injury associated with secondary and tertiary blast effects should be considered when designing and performing neuroimaging studies with patient populations who have suffered blast-related TBI. Depending on the question under investigation, it may be appropriate to exclude subjects with positive anatomical imaging findings consistent with blunt trauma (although limited population numbers may not make this feasible) in an attempt to reduce the relative effects of these injuries on study imaging results. With regard to fMRI, localized injury visible via anatomical imaging will obviously have a significant effect on a subject’s blood oxygen level dependent (BOLD) signal in the injured region and, potentially, regions with which the injured tissue normally communicates. However, the goal of and motivation behind a given study will ultimately dictate how secondary and tertiary blast effects are handled when forming population groups.

## Standard Clinical Imaging is Inadequate to Diagnose Mild TBI

### CT and MRI in moderate to severe TBI

The variety of potential injury mechanisms associated with TBI creates a challenge for identifying the nature and extent of damage in an individual TBI patient. In cases of moderate to severe TBI, anatomical imaging techniques such as CT and standard MRI can provide details about the localization and extent of major injuries such as bone fracture, hematomas, or hemorrhage. Other anatomical sequela such as a shift in the midline of the brain or the presence of a mass effect can also be visible with CT or MRI (Broder, 2010). This structural information about the injury can then be used to assist reparative or neuro-protective procedures (Ling et al., 2009). Jacobs et al. (2011) also found correlations between the amount of midline shift and hematoma volume present and functional outcome 6 months post-injury in a large population of moderate and severe non-blast TBI patients.

### CT and MRI in mild TBI

In cases of mild TBI (blast- and non-blast-related), regions of injury are generally less observable. CT and MRI have been used to identify regions of mild injury to some extent, mostly identifying micro-hemorrhages and contusions (Mittl et al., 1994; McAllister et al., 2001; Benzinger et al., 2009). However, detection of injury-related lesions with these imaging methods has not necessarily led to predictable patient outcome (Scheid et al., 2003; Hughes et al., 2004; Levine et al., 2006). The full extent of DAI is also not always visible via anatomical MRI and CT (Povlishock and Katz, 2005; Levine et al., 2006). Thus, current anatomical imaging techniques may not be sufficient for clinical identification and prognostic evaluation of many mild and some moderate bTBI cases.

## Summary of fMRI

Functional MRI takes advantage of the different magnetic susceptibilities of oxygenated and deoxygenated blood, where signal contrast for a given volume of blood depends on the ratio of deoxygenated to oxygenated blood. For this reason the signal is referred to as a BOLD response. With standard echo-planar-imaging (EPI) sequences, images of the whole brain can be obtained in 2–3 s. This rapid imaging rate, along with the BOLD signal, allows a measure related to blood oxygenation to be made at a relatively good temporal resolution (∼2 s) relative to blood flow changes, and forms the basis of fMRI.

### Hemodynamic response model

In order to determine which areas of the brain are activated by the presented stimuli or task, the time-dependent fMRI signal from each image voxel (3-dimensional “blocks” into which the image space is broken down) is compared to an estimate or model of the signal expected from an ideal activation. The signal model usually takes the form of a prescription for the task or stimuli presentation timing convolved with a hemodynamic response function (HRF). The HRF is the theoretical BOLD response to a single short stimulus. Convolution of this HRF with the time series of the stimuli thus creates a model of the theoretical response of a region of the brain activated by the stimuli. Those voxels with time-courses found to be statistically significantly similar to the modeled stimuli response are characterized as relevantly activated.

### Task design

The specific questions and hypothesis of an investigation play a large role in the design of the task or stimuli presented to the subject. fMRI tasks broadly fall into two categories: (i) block designs and (ii) event-related designs. Studies aimed at investigating a prolonged mental state associated with a given task typically use block designs. Event-related designs, on the other hand, attempt to investigate brain activation in response to individual short-duration events. Generally, a block paradigm is more efficient (i.e., shorter acquisition time), but is constrained by the need for a paradigm that maintains the same mental state for the duration of each block. An event-related paradigm permits additional measurements related to magnitude and shape of the HRF, allowing the estimation of hemodynamic responses associated with individual cognitive processes.

Functional magnetic resonance imaging has been used to investigate many categories of neural processes and responses such as working memory, emotional processing, attention, along with auditory and visual processing. Those with particular relevance to bTBI are discussed below.

## fMRI in Working Memory

Working memory encompasses the mental processes of encoding, storing, manipulating, and retrieving information over relatively short time frames. These processes are essential for most day-to-day tasks and interactions. Difficulty with working memory is a commonly reported impairment for victims of mild to severe blast and non-blast TBI (Dikmen et al., 1986; Alexander, 1995; Vanderploeg et al., 2001; Rosenfeld and Ford, 2010). Results of imaging studies suggest that the neural network associated with working memory is made up of brain regions including areas of the pre-frontal cortex, temporal cortex, parietal cortex, and the cerebellum (Jonides et al., 1998; Smith and Jonides, 1998; D’Esposito et al., 1999a, 2000; Na et al., 2000; Owen, 2000; Ranganath and D’Esposito, 2005; Drobyshevsky et al., 2006).

### Regions associated with aspects of working memory

Results also suggest correlations between certain brain regions and specific functional aspects of working memory. The dorsolateral pre-frontal cortex (DLPFC) has been implicated in assisting with both the manipulation and maintenance of information while the vasolateral pre-frontal cortex (VLPFC) may be more involved with maintenance alone (Smith and Jonides, 1998; D’Esposito et al., 1999b, 2000; Na et al., 2000; Owen, 2000). Regions of the posterior parietal lobe also show evidence of helping to mediate information storage (Jonides et al., 1998; Smith and Jonides, 1998) while regions of the temporal cortex appear to be specifically associated with visual working memory processes (Ranganath and D’Esposito, 2005). There is also evidence to suggest that regions in the left hemisphere are more involved with verbal working memory tasks while the regions in the right hemisphere are more involved with spatial working memory tasks (Smith and Jonides, 1998; Cabeza and Nyberg, 2000; Strangman et al., 2008).

The reliance on multiple regions in a wide range of locations in the brain may make working memory especially susceptible to diffuse TBI-related injuries. Working memory function may be impacted not only by gray matter damage due to hemorrhage or contusion, but also by damage to white matter tracts connecting the various brain regions involved. For instance, damage to the uncinate fasciculus or the superior longitudinal fasciculus could disrupt communication between the frontal lobe and the temporal lobe or the parietal lobe, respectively. Such disruption could slow or limit working memory processes.

### N-back task

Of the various tasks that have been used in fMRI studies to investigate working memory, some of the most common are the N-back task and various forms of a delayed recall (DR) task. In the N-back task, the subject is presented with a rapid series of stimuli and asked to compare each stimulus to one of those previously presented. For each stimulus, the subject must respond whether it is the same or different than the stimulus shown some number (“*N*”) back of previous stimuli. Typically, the N-back task is run with *N* = 1, 2, or 3 stimuli back. A “0-back” version can also be used, in which the subject is given an initial target stimulus and simply responds based on whether each subsequent stimulus is the same or different than the single target stimulus. As the task progresses in the 1-, 2-, or 3-back cases, subjects must continually update and maintain information in working memory. The 3-back level is considered very difficult for most people, taxing the working memory networks to a high degree. There are many variations on the N-back task, including the type of stimuli used (letters, numbers, pictures, etc.) and the method of presentation (visual, auditory, etc.).

The nature of the N-back task allows several points of measurement to be made on the same subject. By increasing N the task can be made more difficult while maintaining the same overall task structure. This allows responses to multiple levels of working memory challenge to be compared within the same subject. The difficulty of the 3-back task should be taken into account when interpreting results, however, as some subjects may exhibit neural activation due to processes not directly related to working memory, such as stress or frustration (Qin et al., 2009).

The N-back task has typically shown activation associated with working memory in the pre-frontal cortex as well as the temporal and parietal cortices in healthy controls (Cohen et al., 1997; Cabeza and Nyberg, 2000; Perlstein et al., 2003). It has also shown differences between controls and non-blast TBI patients (McAllister et al., 1999, 2006; Smits et al., 2009). While both groups were seen to activate similar general regions when carrying out the N-back task, the specific extent of activation corresponding to different levels of working memory challenge differed between healthy controls and TBI patients.

**Figure 1:**
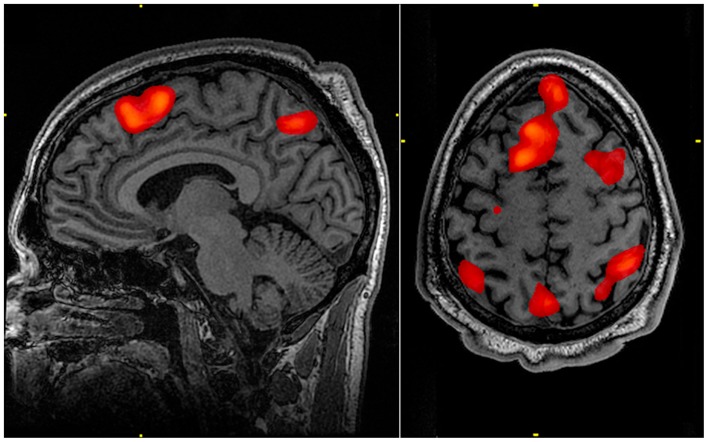
**Working memory tasks typically show activation in the bilateral and superior frontal cortex as well as in parts of the superior bilateral parietal cortex**. The highlighted regions showed significantly different activation between an individual performing a 1-Back task versus a 2-Back task.

Activation of multiple regions across different sections of the brain provides a potential advantage when attempting to investigate the effects and extent of bTBI within an individual. Especially in cases of mild bTBI, where anatomical imaging may not provide a robust localization of injury, the ability to examine multiple areas with a single functional study may provide useful diagnostic information. This examination of multiple neural regions, along with the N-back task’s ability to target various degrees of working memory (Figure 1), makes it a potentially powerful tool for investigating a common functional sequela of bTBI.

### Delayed recall task

Another task often used to investigate working memory is the DR task. The general form of this task involves presenting the subject with a number of stimulus objects to encode, such as a row of letters. The subject maintains the objects in working memory for a short time (on the order of 5–10 s), and is then presented a probe asking the subject to recall one or multiple objects from the initial stimulus group. Alterations to this task can ask the subject to perform mental manipulation of the stimulus objects during the delay period, invoking executive control as well as memory maintenance and recall.

An advantage of the delayed recall task, as with the N-back, is the ability to present varying degrees of working memory challenge (for example, by changing the number of initial stimulus objects to encode). Inclusion of manipulation of the objects held in memory allows a direct within-subject comparison between the encoding-retrieval tasks and the encoding-manipulation-retrieval tasks.

Like the N-back task, DR tasks have been shown to activate various brain regions associated with working memory in healthy controls including the frontal cortex, DLPFC, VLPFC, and parietal regions (D’Esposito et al., 1999a, 2000; Chein and Fiez, 2001; Turner and Levine, 2008). Working memory-related activation has been seen in regions of the cerebellum using DR tasks as well (Desmond et al., 1997; Durisko and Fiez, 2010; Marvel and Desmond, 2010). The task has also been used to show differences in regional activation between healthy controls and subjects with DAI due to motor vehicle accidents (Turner and Levine, 2008). Although the number of subjects is limited (8 patients and 12 controls), DAI patients showed higher task-related activation in several PFC and posterior regions of the brain than did healthy subjects, suggesting that engagement of more of the brain was necessary for the DAI patients to perform the task.

Several characteristics of DR tasks are attractive for investigation of bTBI. Along with the ability to probe the working memory network, the potential to observe more process-specific (encoding, maintenance, manipulation, and retrieval) differences in activation patterns within a subject may help in the localization of mild bTBI and perhaps lead to a better understanding of the subject’s specific functional impairment.

## fMRI in Selective Attention

Many individuals with bTBI also report problems with attention. In general, maintaining attention is the ability to consistently direct mental effort and vigilance toward a target task or goal. This requires the discrimination of related stimuli from unrelated or distracting stimuli as well as control of mental focus. Like working memory, attention is necessary for many day-to-day tasks, especially in a work environment. Impairment of attention due to bTBI can significantly impact quality of life.

### Regions associated with selective attention

The attention neural network consists of several regions of the brain including the DLPFC, ACC, striatum, VLPFC, parietal cortex, and possibly areas of the cerebellum (Bush, 2010). Although the precise role of each of these regions with regard to attention is not fully known, several general trends are suggested in the literature. Areas in the PFC and ACC are thought to be associated with directing cognitive control (Duncan and Owen, 2000; MacDonald et al., 2000; Milham et al., 2003; Haupt et al., 2009). Regions of the dorsal ACC have also been implicated in processes involving response selection and stimulus conflict management (Bush et al., 1998; Bush, 2010). Areas of the parietal cortex involved with attention have mostly been associated with control and direction of visual attention (Corbetta, 1998; Corbetta et al., 2000; Culham and Kanwisher, 2001; Culham, 2002; Bush, 2010). Finally, it has been suggested that the striatum is involved in carrying out behavior related to current situational goals (Haber, 2003).

As with working memory, attention may be affected by TBI through damage to gray matter components of the associated neural network or through damage to white matter tracts that facilitate communication between these components. Axonal injury in the anterior limb of the internal capsule or to the anterior corona radiata may inhibit communication between regions in the frontal cortex and the striata or anterior cingulate.

### Stroop task

A common functional MRI task used to engage and investigate selective attention is the Stroop task (Stroop, 1935; Bush et al., 1998; Carter et al., 2000; Leung et al., 2000; Gruber et al., 2002; Smits et al., 2009). While there is a variety of specific implementations of the Stroop task, the basic principle is to ask the subject to respond to a given characteristic of each presented stimulus while the stimuli themselves exhibit secondary characteristics that are either congruent or incongruent with the characteristic to which the subject is supposed to respond (Stroop, 1935). The classic form of this task is to ask subjects to respond to various words denoting color printed in congruent (for example, the word “red” written in red font) or incongruent (the word “blue” written in red font) font colors. Subjects are asked to either state, as quickly and accurately as they can, the color spelled out by the word or the color text in which the word is written. The incongruous combinations typically lead to longer response times and altered neuronal response patterns. Other implementations involve the subject responding to stimulus characteristics such as the number of words presented at the same time or the relative size of words presented while the words themselves spell out congruent or incongruent number or size characteristics (Bush et al., 1998; Melcher and Gruber, 2009).

Functional MRI studies of healthy adults have shown activations associated with the Stroop task and the response interference condition it presents in areas including the anterior cingulate cortex, medial and inferior frontal lobe, and inferior parietal lobe (Bush et al., 1998; Leung et al., 2000; Gruber et al., 2002; Haupt et al., 2009). The task has also been used to investigate differences between activation associated with selective attention in healthy controls and mild non-blast TBI patients (Smits et al., 2009). A positive correlation was found between increased levels of activation in brain regions involved with selective attention and patients’ reported post concussive symptoms. The Stroop task’s long history of use in a variety of contexts and its focus on a common functional sequela of bTBI make it an attractive diagnostic candidate.

## fMRI in Emotional Response

### Regions associated with emotional response

Victims of bTBI may also show alterations in emotional state. Emotional processing and response have generally been associated with regions of the limbic system, including the amygdala, thalamus, and hippocampus, as well as areas of the medial and inferior PFC and basal ganglia (Van Eden and Buijs, 2000; Phan et al., 2002; Morgane et al., 2005; Drevets et al., 2008). Neuroimaging and other studies have produced results suggesting that some of the regions in this system display activity with emotion-specific correlation. For example, activation in the amygdala seems to be most strongly correlated with fear (Phan et al., 2002; Ohman, 2005), while activation in the basal ganglia seems to be correlated with happiness or disgust (see Phan et al., 2002 for review). However, it is also clear that these correlations do not represent exclusive relationships; activation in the amygdala is seen in emotional responses other than fear (Phan et al., 2002; Zald, 2003; Peper et al., 2006). As with other mental processes, development of a robust anatomy-function relationship model for emotional processing is an ongoing endeavor.

### Emotional response tasks

Functional MRI tasks used to investigate emotion typically present the subject with emotionally charged stimuli and seek to observe elicited changes in brain activity. Stimuli can be in a variety of forms including emotionally charged images, sounds, or even tastes or smells (Small et al., 2003; Drobyshevsky et al., 2006; Koch et al., 2007; Vuilleumier and Pourtois, 2007; Gamer and Buchel, 2009). One of the most commonly used forms of stimuli is emotionally charged faces. Throughout the fMRI task, subjects can be presented with faces expressing a variety of emotions such as happiness, anger, fear, or concern. Using photo manipulation software, the relative degree to which the faces epitomize these emotions can also be changed (Lennox et al., 2004). Thus, the presentation of emotional faces allows the subject to be tasked with both a wide range of emotions as well as a range of the degree of these emotions. Such tests have been used in the investigation of various mood disorders, including bipolar disorder and major depressive disorder (MDD; Lennox et al., 2004; Surguladze et al., 2005; Leppanen, 2006).

The emotional Stroop task has also been used to investigate alterations in response to emotionally charged words. As with the classic Stroop task, in the emotional Stroop subjects are asked to respond to a characteristic of presented words, such as the number of words shown at once or the color in which the words are presented. In the emotional variant of the task, however, words of various emotional valences are used and subjects’ performances in response to various categories of words are compared; for example, performance in response to generally positive words (“gift,” “love,” etc.) may be compared to performance in response to generally negative words (“hate,” “murder,” etc.). Williams et al. (1996) provide a review of many applications of the emotional Stroop, showing behavioral differences in a range of anxiety, depressive, and other psychological conditions. Recent imaging studies using PET and fMRI have also shown functional activation differences related to emotional category of stimulus word presentation in patients with PTSD (Shin et al., 2001; Bremner et al., 2004; McNally, 2006). While the emotional Stroop task may provide information on brain regions affected by bTBI, its sensitivity to psychological disorders highlights the fact that these must be taken into consideration when designing and interpreting results from fMRI studies using this task.

### Emotional response and bTBI

The relationship between bTBI and the alteration of emotional processing is far from apparent. Conclusive findings have yet to be made tying region-specific injury to emotional consequences (Schwarzbold et al., 2008). Furthermore, the high degree of comorbidity between bTBI and PTSD and MDD presents an additional challenge to accurate diagnosis (Elder et al., 2010).

## Resting State fMRI

The previous sections have described task-oriented fMRI techniques, which require the subject to observe and respond to various stimuli while in the scanner. In contrast to these study designs, resting state fMRI is performed as the subject simply rests in the scanner. Even when “resting,” the human brain is not inactive. When the subject is resting in the scanner, fluctuations are seen in the BOLD signal. For a time these fluctuations were considered to be noise by many researchers, but close inspection has revealed that they are not entirely random. Specifically, various regions of the brain may show very similar fluctuation patterns. Resting state fMRI takes advantage of these spontaneous fluctuations to investigate connectivity within the brain (for an in-depth review, the reader is referred to Fox and Raichle, 2007).

The link between shared spontaneous BOLD activity and functional connectivity is the idea that brain regions involved in the same mental process will exhibit similar activity patterns, even in a “resting” state. Thus, coherence in spontaneous BOLD fluctuations of different regions may provide an indirect measure of network connectivity. This idea is supported by results in the literature showing that spontaneous BOLD fluctuations can indeed be used to identify several well-known networks, including the somatomotor system and the visual system (Lowe et al., 1998; Cordes et al., 2000; De Luca et al., 2005; Fox and Raichle, 2007).

### Default mode network

**Figure 2:**
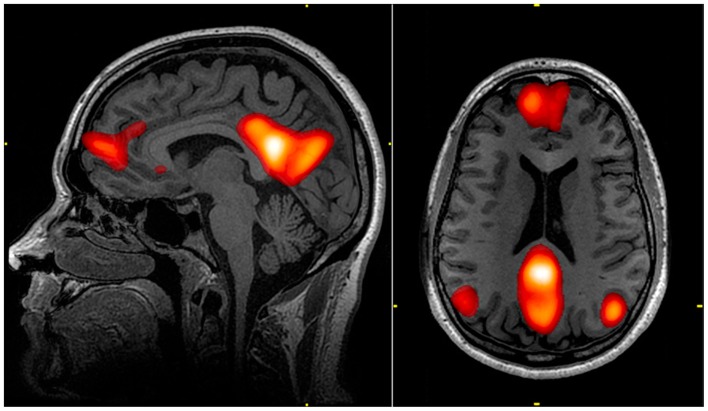
**The default mode network (DMN) includes regions in the medial pre-frontal cortex, precuneus, and bilateral parietal cortex**. Highlighted regions in the images were all correlated with the same component from an independent component analysis of an individual’s resting state fMRI data. The color scale reflects correlation *z*-scores, with a lower threshold of *z* = 3.

Raichle et al. (2001) described a “default mode network” (DMN) within the brain. The regions in this network exhibit decreased activation associated with many goal-oriented or attention-demanding tasks and were therefore proposed to facilitate a “default” functional state within the brain. This network includes the medial pre-frontal cortex, precuneus, posterior cingulate, and bilateral parietal lobe (Raichle et al., 2001; Figure 2). Various regions of the DMN may be responsible for processes such as introspective or self-referential thought, monitoring of the external environment, emotional processing (Gusnard et al., 2001; Broyd et al., 2009), spontaneous cognition, and predicting possible actions demanded by the environment (Raichle and Snyder, 2007). Dysfunction of the DMN may contribute to functional deficits (Broyd et al., 2009). Weissman et al. (2006) found that less task-induced deactivation of regions in the DMN correlated with increased response time in healthy subjects performing an attention task. Sonuga-Barke and Castellanos (2007) presented a “default mode interference hypothesis” which suggests that inappropriate activation of the DMN during attention-demanding tasks may detract from mental effort put toward the task, leading to distraction and poorer behavioral performance. It is possible that damage to the DMN due to bTBI could bolster such inappropriate spontaneous activation and lead to attention-deficits often seen in bTBI victims.

### Applications of resting state fMRI

Resting state fMRI connectivity analysis has been applied to several neurological disorders and diseases (Voss and Schiff, 2009) including Parkinson’s Disease (Wu et al., 2009; Helmich et al., 2010), schizophrenia (Ke et al., 2010; Skudlarski et al., 2010), MDD (Anand et al., 2005a,b; Greicius et al., 2007), attention-deficit hyperactivity disorder (Tian et al., 2006; Kelly et al., 2007; Castellanos et al., 2009), Alzheimer’s Disease (Li et al., 2002; Supekar et al., 2008; Koch et al., 2012), and post-traumatic stress disorder (Bluhm et al., 2009b). In most cases, evidence was found of a relationship between disease state and network connectivity in brain regions believed to be associated with the disease under investigation. Nakamura et al. (2009) have analyzed resting network plasticity in a small group of TBI patients, showing change in network characteristics over a period of recovery. The capacity to correlate network connectivity with behavioral sequelae presents an important potential use of resting state fMRI in the investigation of bTBI.

### Advantages for the study of bTBI

Resting state fMRI studies also present several logistical advantages when investigating a patient population such as bTBI victims (Fox and Greicius, 2010). Resting state fMRI does not depend on the subject’s ability to perform a given task. This may be particularly relevant to blast-related TBI populations, where rates of comorbidity with other major injuries such as limb loss may be high. Such major injuries may preclude a subject from performing a task requiring a specific motor response. Resting state fMRI studies also reduce the number of possible complicating factors when attempting to replicate protocols across sites, reducing variability in multi-site studies and assisting in the accumulation of larger numbers of potentially comparable sets of patient data.

In addition to these logistical advantages, resting state connectivity analysis is not limited to investigating a single functional network but allows the identification of multiple brain networks (De Luca et al., 2006; Mantini et al., 2007; Voss and Schiff, 2009). This ability to interrogate the connectivity of multiple networks simultaneously could be very powerful in locating specific injuries in individuals within a bTBI population.

## Oculomotor Tasks and TBI

Vestibular symptoms, such as dizziness, are also common following bTBI. Several recent studies have shown the potential for visual oculomotor tasks and testing to be sensitive to the presence of blast and non-blast-related TBI (Suh et al., 2006; Kraus et al., 2010; Scherer et al., 2011). In these studies, poor or altered performance on oculomotor tasks correlated with the presence of TBI or increased TBI symptom severity.

Oculomotor tasks used in combination with fMRI, EEG, and PET imaging studies have helped identify regions of the brain associated with performance of eye saccades (for in-depth review, see McDowell et al., 2008). As with the more complex mental processes mentioned above, the generation of eye saccades involves areas spread across the brain, including occipital cortex, lateral frontal cortex, and parietal cortex, as well as subcortical regions including the striatum, thalamus, and regions of the cerebellum. Furthermore, more complex eye movement, such as predictive saccades, may involve more frontal brain regions (Simo et al., 2005; McDowell et al., 2008). Thus, performance of eye saccades, especially those of greater complexity, may be hindered as a result DAI due to bTBI.

Two of the studies mentioned above (Suh et al., 2006; Kraus et al., 2010) specifically show alteration in predictive eye movement (where the visual stimuli are presented in a learnable pattern) in subjects with non-blast-related TBI, suggesting the presence of frontal lobe damage or white matter damage between regions of the brain associated with predictive saccade performance. Although these studies did not involve subjects with bTBI, predictive saccade tasks show promise for being sensitive to the presence of TBI.

Functional magnetic resonance imaging shows potential as a means of confirming the neurophysiological underpinnings of the sensitivity of predictive saccade tasks to the presence of bTBI. Previous work has already shown differences between fMRI activation patterns elicited by a predictive saccade task and a visually guided saccade task (in which the visual stimuli appear randomly, thus excluding any predictive learning element from the task) in healthy controls (Suh et al., 2006). Similar studies involving bTBI subjects as well as healthy controls may provide imaging results that support the current behavioral findings.

## Comorbidity of PTSD and Major Depressive Disorder with mTBI and Considerations for fMRI

Post-traumatic stress disorder and MDD have been increasingly common in U.S. soldiers returning from current conflicts (Tanielian and Jaycox, 2008). While a causal relationship between mild TBI and PTSD or MDD has not been identified, several studies have shown correlations between mTBI and these psychological health issues in military populations returning from deployment (Hoge et al., 2008; Schneiderman et al., 2008; Vanderploeg et al., 2012; Bazarian et al., 2013). Correlations between TBI and MDD have also been reported in non-military populations (Jorge et al., 2004; Kim et al., 2007; Bombardier et al., 2010). Vanderploeg et al. (2012) found that injury due to blast did not significantly increase risk of developing PTSD or MDD over injury due to other mechanisms in a National Guard population, but this finding has not been consistent in other populations. And, while concurrent diagnosis of bTBI and PTSD is difficult due to common symptoms and problems with self-report assessments (Capehart and Bass, 2012), there may still be relatively high rates of PTSD or MDD within a population of patients who have experienced bTBI.

Several previously published reviews highlight both functional and anatomical neuroimaging findings associated with PTSD and MDD (Leppanen, 2006; Bremner, 2007; Lorenzetti et al., 2009; Holzschneider and Mulert, 2011; Hughes and Shin, 2011; Robinson and Shergill, 2011; Stuhrmann et al., 2011; Delvecchio et al., 2012). The findings that most directly relate to the application of fMRI to individuals with comorbid bTBI and PTSD or MDD involve alterations in brain activation patterns attributable solely to these two disorders. Various emotionally charged or trauma-related tasks used in previous fMRI studies of PTSD have revealed activation alterations in regions including the hippocampus and parahippocampal gyrus, ACC, thalamus, medial frontal gyrus, amygdala, mPFC, and ventral frontoparietal region (Lanius et al., 2001, 2003; Shin et al., 2001, 2005; Yang et al., 2004; Sakamoto et al., 2005). Several previous fMRI studies of MDD using emotional stimuli have shown activation differences associated with MDD in the amygdala (Siegle et al., 2002; Surguladze et al., 2005; Leppanen, 2006; Stuhrmann et al., 2011). Activation alterations associated with MDD in response to emotional facial stimuli have also been reported in regions including the insula, parahippocampal gyrus, putamen, caudate, and orbitofrontal cortex (Stuhrmann et al., 2011; Delvecchio et al., 2012). Directly relevant to bTBI, Matthews et al. (2011) found differences in amygdala activation associated with the presence of MDD in a population consisting of subjects who had all experienced bTBI; differences in amygdala response were found when comparing subjects with bTBI and MDD to those who only had bTBI.

Differences in activation patterns between control subjects and subjects with PTSD have also been seen in response to emotionally neutral, non-trauma-related fMRI tasks. Using an auditory oddball paradigm (in which subjects were asked to provide a motor response to relatively infrequent target tone stimuli interspersed within more frequent standard tone stimuli), Bryant et al. (2005) saw increased ACC and left amygdala activation in PTSD subjects relative to controls. Moores et al. (2008) reported abnormal activation associated with PTSD in bilateral DLPFC and inferior parietal lobe during the memory maintenance period of a working memory task. During the memory updating periods of this task the PTSD group showed relatively less activation in hippocampus, anterior cingulate, and brainstem pons. Astur et al. (2006) also reported a negative correlation between PTSD severity and hippocampal activation when subjects performed an fMRI adaptation of a water maze task. Finally, Geuze et al. (2008) reported that, during performance of a neutral word-pair encoding and retrieval task subjects with PTSD showed less activation in the frontal cortex and greater activation in the temporal cortex relative to controls while encoding information, and less activation in the right frontal cortex, bilateral middle temporal gyri, and left posterior hippocampus relative to controls while retrieving information.

The presence of PTSD or MDD has also been shown to correspond to alterations in resting state fMRI data. Studies have reported PTSD-related changes in resting state connectivity between regions of the limbic system and cortical regions or between the PCC and cortical or subcortical regions. A seed-based analysis performed by Yin et al. (2011) showed group differences in connectivity measures between the thalamus and cortical regions including frontal gyri and ACC when comparing a PTSD patient group with healthy controls. Two studies comparing combat veterans with and without PTSD reported increased connectivity between the amygdala and the insula associated with PTSD (Rabinak et al., 2011; Sripada et al., 2012). Sripada et al. (2012) also found decreased connectivity measures between the amygdala and the hippocampus in the PTSD group. Studies looking specifically at connectivity with the PCC have found PTSD-related connectivity alterations between this region and a number of other regions throughout the brain, including parts of the occipital, temporal, and frontal lobes, the insula, the ACC, the amygdala, and the hippocampus (Bluhm et al., 2009a; Qin et al., 2012).

Resting state studies of MDD have reported a variety of changes in connectivity associated with MDD. While, in general, these studies do not seem to reach a consensus about functional connectivity changes introduced by MDD, several show changes in the DMN. A recent study by Zhu et al. (2012) that investigated changes in the functional connectivity of the DMN showed increased connectivity in the mPFC and ACC and decreased connectivity in the PCC with the rest of the DMN in patients with MDD. Using seed regions based on MDD-related changes in gray matter volume, Ma et al. (2012) found alterations in resting state functional connectivity between the middle temporal gyrus and portions of the DMN and between the right caudate and portions of the frontal cortex associated with MDD, while Guo et al. (2012) saw decreased cross-hemispheric signal correlation in the mPFC and PCC in patients with MDD. Other resting state studies investigating MDD have shown altered connectivity between other cortical and subcortical regions (Anand et al., 2005a; Cao et al., 2012; Tang et al., 2012; Ma et al., 2013). Finally, Horn et al. (2010) found increased functional connectivity between the pregenual ACC and the anterior insula in a subset of severely depressed patients.

Given the rates of comorbidity of PTSD and MDD with mTBI and the effects these disorders can have on brain activation patterns in response to a variety of fMRI tasks, measurements of PTSD and MDD presence or severity [such as the PTSD checklist (Weathers et al., 1993) and the Beck Depression Inventory 2 (Beck et al., 1996)] should be taken on subjects involved in bTBI fMRI studies. In a military population, the PTSD checklist (PCL-C or PCL-M; Weathers et al., 1993) is frequently used, as is the Beck Depression Inventory 2 (Beck et al., 1996). In some cases, a more definitive and careful assessment of PTSD, such as the CAPS (Clinician-Administered PTSD Scale) should be considered. These measures should then be included in the analysis model as possible explanations of variance in activation or connectivity measures.

## Statistical Considerations for fMRI Relevant to bTBI Populations

Over the past decade fMRI has become increasingly sophisticated and has been used to investigate increasingly complex questions about the nature of human neural function. Proper interpretation of fMRI data, however, relies upon statistically robust data acquisition and analysis. This dependency stems from the relatively small BOLD signal (changes on the order of 1–5%) which is invariably accompanied by several sources of noise. Since an fMRI experiment is typically composed of multiple measures compared to an ideal model, the signal as well as the noise is difficult to estimate. Furthermore, most fMRI results are not a simple magnitude or effect size, but rather are cast in terms of statistical reliability. Care must be taken when interpreting statistical results, as this will obviously affect any neuro-functional conclusions.

### Group analysis considerations

Given the inherent heterogeneity of bTBI and traumatic brain injury in general, there are several important considerations to be made when interpreting group analysis results. Several papers have addressed the effects of inhomogeneous activation within populations consisting of healthy subjects These effects may be even greater in TBI populations.

Thirion et al. (2007) present an analysis of the reliability of group-wise activation maps for an event-related study design. They define reliability in two ways: stability of each voxel with regard to its identity as active or inactive, and how frequently significant clusters appear within the same locations across the subjects in a population. Working with data sets from 78 subjects they break the population into smaller groups of various sizes and compare the results from each sub-group for each sub-group size. The stability of voxel identity and agreement between cluster locations within a sub-group increased as the size of the sub-groups was increased. Their results suggest the need for between 20 and 25 subjects within a defined homogenous group for reliable statistical analyses of this event-related study design (Thirion et al., 2007). Along the same lines, Friston et al. (1999) have shown that, for an appropriately sensitive test, close to 20 conjunctional subject results are needed for a statistically reliable determination that 85–90% of a population will exhibit that result.

Murphy and Garavan (2004) also presented empirical work investigating the relative effect of group analysis size on statistically significant results from a GO/NO-GO event-related study design. In this work, they compared the statistical activation results from an analysis of a full group of 58 healthy volunteers to sub-groups of various sizes. Their data showed a need for about 50 subjects in the sub-group to obtain 80% power (defined by the percent of voxels seen in the full group activation map also seen in the sub-group activation map) for *p*-value thresholds ranging from 0.000001 to 0.001. However, even at smaller sub-group levels the majority of voxels reported as activated in the sub-groups were also seen as activated in the full group activation map. With a sub-group size of 20 subjects, 80% of the activated voxels seen in the sub-group activation map were also seen in the full group activation map for *p*-value thresholds from 0.01 to 0.0001. This behavior led to the conclusion that the sub-group activation maps contained regions of false negative voxels (i.e., loss of sensitivity) but not necessarily a large number of false-positive voxels (Murphy and Garavan, 2004; i.e., specificity is not greatly hampered).

Desmond and Glover (2002) present a statistical power analysis of a simulated block study design. They take into consideration the variability in the fMRI signal due to both inter- and intra-subject sources, the size of the experimental effect seen in the fMRI data, the number of time points per block condition, and the number of subjects. With an experimental effect size of 0.5%, intra- and inter-subject variability sizes of 0.75 and 0.5%, respectively, and a desired alpha value of 0.05, it was found that 12 subjects were needed to maintain 80% statistical power (Desmond and Glover, 2002). Keeping all other variables the same, changing the desired alpha to a more conservative 0.002 necessitated 21–22 subjects to maintain 80% power. Changes in inter-subject variability (which may be particularly relevant to bTBI) also obviously affected the number of subjects required to maintain statistical power. With an increase of this variability from 0.5 to 0.7%, the number of subjects needed for 80% power at an alpha of 0.05 increased from 12 to approximately 20.

These papers highlight statistical issues that are especially pertinent to group analysis in bTBI studies. First, group size will have a large impact on the reliability of group-wise statistical maps. One of the common potential weaknesses in current fMRI TBI study literature is the relatively low number of patients in each study. The arguments presented above show that extreme caution must be taken when interpreting group analysis results from small groups, especially when comparing patient group results with results from a control population of a different size. The papers discussed above that deal with event-related study design suggest that groups should consist of at least 20 subjects for reliable group parametric maps. Under optimal conditions fewer subjects may be needed to maintain statistical power using block study designs, but characteristics of the study including the relative size of the contrast being observed and sources of possible variability within the subject population should be taken into consideration when interpreting group analysis results.

Second, the major underlying factor contributing to the various group activation map disparities analyzed in the papers focused on event-related designs (Murphy and Garavan, 2004; Thirion et al., 2007) is differences in the individual subject activation maps, leading to heterogeneity in the group pools. Spatial heterogeneities were found in the activation results even in populations of healthy subjects performing relatively simple fMRI tasks: visual checkerboard, cued left-right button presses, computation, and sentence processing (Thirion et al., 2007); Go/No-Go (Murphy and Garavan, 2004). The level of inter-subject variability also had a large effect on the number of subjects needed to maintain statistical power in the block design study simulations performed by Desmond and Glover (2002). When considering a patient population with a potentially heterogeneous injury mechanism such as bTBI, and performing more complicated tasks investigating more subtle activation, these effects will most likely be exacerbated, leading to fairly unreliable group activation maps, maps that have regions of false negatives, or low statistical power. In these cases, the classic approach of using a voxel-wise group map for summary statistics will yield suboptimal results.

### Interpretation of results in the presence of comorbidities

The high rate of comorbid conditions with bTBI must be considered when interpreting results from fMRI studies of bTBI populations. Factors that may affect individuals’ performance of fMRI tasks or have a reasonable chance of affecting activity or resting state connectivity estimates in regions of interest should be taken into account in group data analysis models to avoid false-positive results or incorrect conclusions about what is driving observed group differences. Carefully designed exclusion/inclusion criteria may also help avoid these issues.

## Conclusion

Given the nature of current military conflicts, bTBI has become an increasing occurrence in returning soldiers. At the same time, little is known about the precise neurobiological effects of primary blast-waves on the brain, and current clinical imaging methods cannot adequately diagnose the injury, especially in mild cases. Both task-based and resting state fMRI have the potential to provide useful objective diagnostic information associated with functional sequelae, including problems with attention, memory, and emotional processing, commonly reported in bTBI victims. Given the generally heterogeneous nature of bTBI and high rates of comorbidity with PTSD and MDD, additional considerations must be kept in mind when composing and interpreting group analysis results. Future studies should include sufficiently large (>20) patient populations and matched numbers of controls. Measures of group data heterogeneity and susceptibility to outliers would also support interpretations of group results.

## Conflict of Interest Statement

The authors declare that the research was conducted in the absence of any commercial or financial relationships that could be construed as a potential conflict of interest.

## Footnotes

^1^http://www.dvbic.org/TBI-Numbers.aspx

^2^www.ncbi.nlm.nih.gov/pubmed/

## Author Note

We certify that all individuals who qualify as authors have been listed; each has participated in the conception and design of this work, the writing of the document, and the approval of the submission of this version; that the document represents valid work; that if we used information derived from another source, we obtained all necessary approvals to use it and made appropriate acknowledgments in the document; and that each takes public responsibility for it. Nothing in the presentation implies any Federal/DOD/DON endorsement.
